# Down-regulation of HPGD by miR-146b-3p promotes cervical cancer cell proliferation, migration and anchorage-independent growth through activation of STAT3 and AKT pathways

**DOI:** 10.1038/s41419-018-1059-y

**Published:** 2018-10-17

**Authors:** Shuihong Yao, Jingyun Xu, Kaixuan Zhao, Pengxia Song, Qin Yan, Weifei Fan, Wan Li, Chun Lu

**Affiliations:** 1 State Key Laboratory of Reproductive Medicine, Nanjing Medical University, Nanjing, 211166 P. R. China; 2 Medical School, Quzhou College of Technology, Quzhou, 324000 P. R. China; 3 Key Laboratory of Pathogen Biology of Jiangsu Province, Nanjing Medical University, Nanjing, 211166 P. R. China; 4 Department of Microbiology, Nanjing Medical University, Nanjing, 211166 P. R. China; 5 Department of Hematology and Oncology, Department of Geriatric Lung Cancer Research Laboratory, Geriatric Hospital of Nanjing Medical University, Nanjing, 210024 P. R. China

## Abstract

While the application of early screening and HPV vaccines has reduced the incidence and mortality rates of cervical cancer, it remains the third most common carcinoma and fourth leading cause of cancer-associated death among women worldwide. The precise mechanisms underlying progression of cervical cancer are not fully understood at present. Here, we detected significant down-regulation of 15-hydroxyprostaglandin dehydrogenase (HPGD) in cervical cancer tissues. Overexpression of HPGD inhibited cervical cancer cell proliferation, migration and anchorage-independent growth to a significant extent. To clarify the mechanisms underlying HPGD down-regulation in cervical cancer, miRNA microarray, bioinformatics and luciferase reporter analyses were performed. HPGD was identified as a direct target of miR-146b-3p displaying up-regulation in cervical cancer tissues. Similar to the effects of HPGD overexpression, down-regulation of miR-146b-3p strongly suppressed proliferation, migration and anchorage-independent growth of cervical cancer cells. Furthermore, HPGD negatively regulated activities of STAT3 and AKT that promote cervical cancer cell proliferation. Notably, HPV oncogenes E6 and E7 were determined as potential contributory factors to these alterations. Our results collectively suggest that the HPGD/miR-146b-3p axis plays a significant role in cervical cancer and may serve as a potentially effective therapeutic target.

## Bullet Points

- HPGD inhibits cervical cancer cell proliferation, migration and anchorage-independent growth by negatively regulating STAT3 and AKT pathways.
- miR-146b-3p directly targets HPGD.
- Downregulation of HPGD and up-regulation of miR-146b-3p in cervical cancer are mediated by the high-risk HPVs E6 and E7.

## Introduction

Cervical cancer, one of the most common gynecological tumors, is the fourth leading cause of cancer-associated deaths among women worldwide^1^. Cancer arising from the cervix is initially asymptomatic, later transforming into high-grade squamous intraepithelial lesions and invasive cancer^2^. A number of proteins reported to be abnormally expressed in cervical cancer may serve as potential prognostic markers and therapeutic targets for treatment, including Wnt family member 5A (Wnt5A)^3^, ATPase family AAA domain-containing protein 2 (ATAD2)^4^, sphingosine kinase 1^5^ and erythropoietin-producing human hepatocellular carcinoma receptor B2 (EphB2)^6^. Transcription factor sex-determining region Y-box 9 protein (SOX9) is known to suppress cervical tumor growth through binding a specific promoter region of p21 (WAF1/CIP1) to block G1/S transition^7^. Neuroblastoma breakpoint family member 1 (NBPF1) inhibits cervical cancer invasion via regulating the PI3K/mTOR signaling pathway^8^.

Human papillomavirus (HPV) infection is predominantly responsible for the incidence of cervical cancer^9^. According to statistical analyses, among the 15 high-risk HPV types, HPV-16 and HPV-18 are responsible for 75% cervical cancer cases^10,11^. The HPV genome integrates into the host genome following which the viral oncogenes are constitutively expressed^12^. HPV oncoproteins E6 and E7 that are consistently overexpressed in cervical cancers activate the phosphoinositide 3-kinase (PI3K)/protein kinase B (Akt) and Wnt/β-catenin/Notch pathways, inducing significant tumorigenic transformation^13^. Persistent infection of high-risk HPVs is necessary, but not sufficient to cause cervical cancer^14–17^. Additionally, long-term use of oral contraceptives^18^, certain sexually transmitted infections^19^, early sexual exposure, multiple partners, high parity, and smoking^20^ may contribute to the relative risk of developing cervical cancer.

15-Hydroxyprostaglandin dehydrogenase (HPGD) has been identified as a tumor suppressor in various malignancies, including gastrointestinal^21^, bladder^22^ and lung cancers^23^. Prostaglandins are involved in tumor progression through modulation of angiogenesis, migration, invasion, proliferation, and apoptosis^24^. A key enzyme participating in the metabolism of prostaglandins^25^, HPGD regulates their bioavailability through conversion into the biologically inactive forms, 13,14-dihydro-15-keto PGF2 and 13,14-dihydro-15-keto PGE2^26–28^. HPGD additionally has the ability to inhibit prostaglandin-endoperoxide synthase 2^21,29–32^, which catalyzes the metabolic conversion of arachidonic acid to prostaglandins^33,34^. While HPGD is clearly implicated in several malignancies, its potential association with cervical cancer development is unknown at present.

In this study, we analyzed cervical cancer and paracancerous tissues of patients using gene microarray. HPGD was significantly down-regulated in cervical cancer tissues, and conversely, overexpression of HPGD associated with dramatically reduced cancer cell proliferation, migration and anchorage-independent growth. miR-146b-3p, which directly targets HPGD, was up-regulated in cervical cancer tissues, contributing to enhanced cell proliferation, migration and anchorage-independent growth. Moreover, HPGD suppressed activation of STAT3 and AKT pathways and these alterations were potentially mediated by HPV oncoproteins E6 and E7.

## Results

### HPGD is down-regulated in cervical cancer tissues

**Fig. 1:**
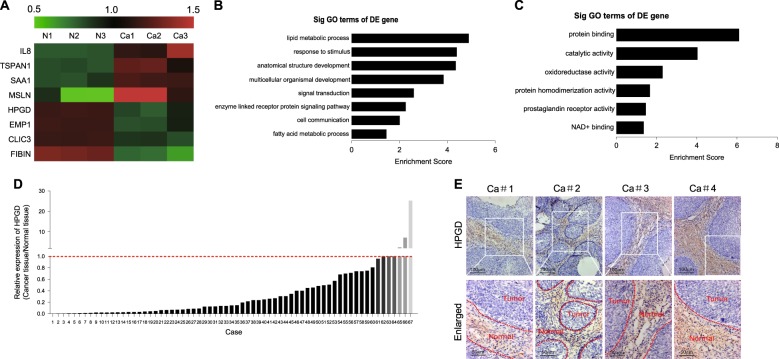
HPGD is down-regulated in cervical cancer tissues (**a**) A representative result of cervical cancer gene microarray analysis. (**b, c**) GO analysis of HPGD. (**d**) mRNA expression of HPGD in 67 pairs of cervical cancer and adjacent non-cancerous tissues determined via qPCR. (**e**) Immunohistochemical (IHC) staining of HPGD in cervical cancer tissues

To identify the critical genes correlated with cervical cancer development, three pairs of cervical cancer and paracancerous tissues were analyzed via gene microarray. In total, 990 genes were up-regulated and 1446 genes down-regulated by more than two fold in cervical cancer, compared with paracancerous tissues. Some of the altered genes are presented via hierarchical clustering (Fig. 1a). Among these, HPGD, known to be significantly associated with cancer development, drew our attention. Data from GO analyses indicated that HPGD is closely related to metabolic process, response to stimulus, protein binding, and catalytic activity (Fig. 1b, c). Validation was performed using 67 pairs of cervical cancer and adjacent non-cancerous tissues. As shown in Fig. 1d, in the majority of cervical cancer tissue specimens (60/67), HPGD expression was significantly lower relative to paracancerous tissues. Immunohistochemical staining consistently revealed lower numbers of HPGD-positive cells in cervical cancer than normal tissues (Fig. 1e).

### HPGD overexpression inhibits cell proliferation, migration and anchorage-independent growth

**Fig. 2:**
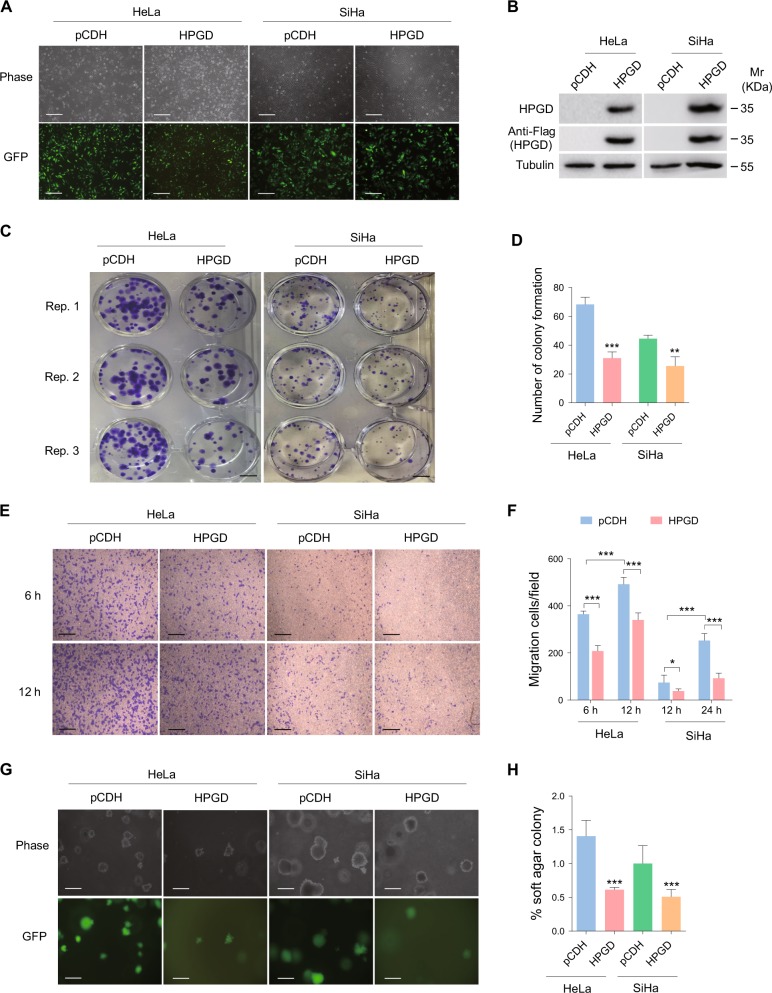
HPGD overexpression inhibits cell proliferation, migration and anchorage-independent growth (**a**) HeLa and SiHa cells were transduced with lentivirus empty vector (**pCDH**; left) and lentivirus-HPGD (**HPGD**; right), with representative images obtained under a light microscope (**Phase**; top) and fluorescent microscope (**GFP**; bottom) (Original magnification, × 100). Scale bar, 40 μm. (**b**) Western blot performed on cells treated as in (**a**) with the indicated antibodies. (**c**) Plate colony formation assay of cells treated as in (**a**). Scale bar, 1 cm. (**d**) Quantification of cell colony formation described in (**c**). (**e**) Transwell migration assay of cells treated as in (**a**). Scale bar, 40 μm. (**f**) Quantification of the results in (**e**). (**g**) Soft agar colony formation assay of cells treated as in (**a**). Scale bar, 40 μm. (**h**) Quantification of the results in (**g**). Quantified results represent means ± SD. Three independent experiments, each with quartic technical replicates, were performed. ** *P < *0.01 and *** *P* < 0.001 for Student's t-test

In view of the decreased expression of HPGD in cervical cancer tissues, the cervical cancer cell lines, HeLa and SiHa, were transduced with lentivirus-HPGD (Fig. 2a, b), and the cell colony formation assay conducted. As shown in Fig. 2c, d, overexpression of HPGD induced a significant reduction in cell proliferation, compared with the control cell group. In the transwell migration assay, HPGD-infected HeLa and SiHa cells exhibited lower migration ability relative to control cells (Fig. 2e, f). The soft agar colony formation assay was adopted to determine the influence of HPGD on tumorigenicity *in vitro*. Notably, HPGD dramatically suppressed anchorage-independent growth of cervical cancer cells (Fig. 2g, h). Considering the association of HPGD with fatty acid metabolic processes in GO analyses, we further determined the expression patterns of four enzymes involved in *de novo* synthesis of fatty acids, specifically, ATP Citrate Lyase (ACLY), Fatty Acid Synthase (FAS), Fatty Acyl-CoA Elongase 6 (ELOVL6), and Stearoyl-CoA Desaturase-1 (SCD1), in HPGD-transduced HeLa and SiHa cells via RT-PCR. Transcription levels of all four fatty acid-related enzymes were down-regulated in the presence of HPGD, in strong agreement with GO data (Supplementary Figure 1).

### HPGD is a direct target of miR-146b-3p

**Fig. 3:**
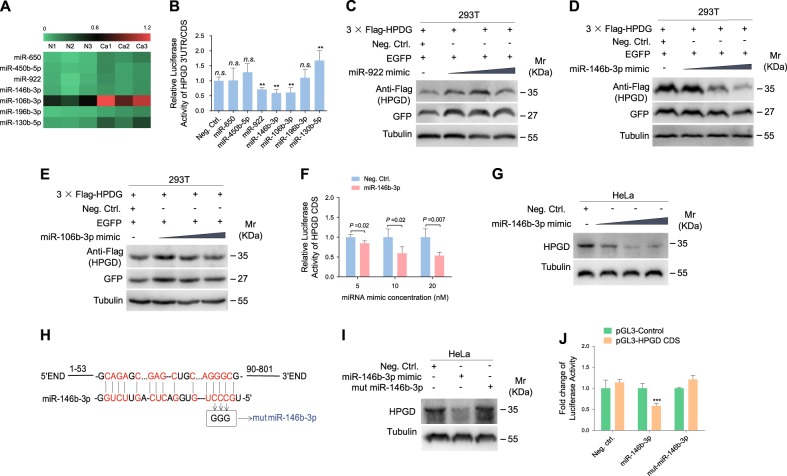
HPGD is directly targeted by miR-146b-3p. (**a**) Representative results of cervical cancer miRNA microarray analysis. (**b**) Luciferase assay of HEK293T cells co-transfected with negative control miRNA (Neg. Ctrl.) or mimics of several miRNAs together with the pGL3-HPGD 3' UTR or pGL3-HPGD CDS luciferase reporter. (**c**) 293T cells were co-transfected with the 3 × Flag-HPGD construct and miR-922 mimic for 48 h. Cells were collected and immunoblotted with the indicated antibodies. (**d**) HEK293T cells were co-transfected with the 3 × Flag-HPGD construct and mimic of miR-146b-3p for 48 h. Cells were collected and immunoblotted with the indicated antibodies. (**e**) HEK293T cells were co-transfected with the 3 × Flag-HPGD construct along with pEGFP and a mimic of miR-106b-3p for 48 h. Cells were collected and immunoblotted with the indicated antibodies. (**f**) Luciferase assay of HEK293T cells co-transfected with pGL3-HPGD CDS reporter together with increasing amounts (5, 10, and 20 nM) of negative control miRNA (**Neg. Ctrl**.) or a mimic of miR-146b-3p (**miR-146b-3p**) for 24 h. (**g**) HeLa cells were transfected with increasing amounts (10, 20 and 50 nM) of miR-146b-3p mimic or negative control for 48 h, collected, and western blot performed with the indicated antibodies. (**h**) Putative binding site of miR-146b-3p in the CDS region of HPGD and mutagenesis of the target site in miR-146b-3p. (**i**) Western blot of HPGD expression in HeLa cells transfected with a negative control mimic (**Neg. Ctrl**; 20 nM), miR-146b-3p mimic (**miR-146b-3p**; 20 nM) or miR-146b-3p mutant mimic (**miR-146b-3p mut**; 20 nM) for 48 h. (**j**) Luciferase activity in HEK293T cells co-transfected with miR-146b-3p mimic (**miR-146b-3p**; 10 nM), miR-146b-3p mutant mimic (**miR-146b-3p mut** ; 10 nM) or a negative control (**Neg. Ctrl**; 10 nM) and the HPGD CDS reporter construct for 48 h. The quantified results are presented as means ± SD. Three independent experiments, each with four technical replicates, were performed. ** *P < *0.01 for Student's t-test. *n.s*, not significant

To identify the miRNAs directly targeting HPGD, we used microarray-based miRNA expression profiling for assessing differentially expressed miRNAs in cervical cancer and paracancerous tissues. Among the miRNAs up-regulated in cervical cancer tissues, seven were predicted to contain putative target sites in the 3' UTR or CDS of HPGD using bioinformatics analysis (Fig. 3a), which were selected for the luciferase reporter assay. As shown in Fig. 3b, miR-922, miR-146b-3p and miR-106b-3p significantly inhibited HPGD CDS reporter activity. To directly confirm the effects of these three miRNAs on HPGD protein, HEK 293T cells were co-transfected with miRNA mimics and the HPGD expression plasmid pCDH-HPGD CDS. Western blot experiments showed that only miR-146b-3p attenuated expression of HPGD in a dose-dependent manner (Fig. 3c–e). Additionally, miR-146b-3p decreased the CDS reporter activity of HPGD (Fig. 3f) and suppressed endogenous HPGD expression (Fig. 3g) in a dose-dependent manner. Mutagenesis of miR-146b-3p was subsequently conducted to further validate whether HPGD is a direct target (Fig. 3h). The mutant mimic did not affect CDS reporter activity (Fig. 3i) or protein levels of HPGD (Fig. 3j), confirming that miR-146b-3p directly targets HPGD.

### miR-146b-3p inhibition attenuates cell proliferation, migration and anchorage-independent growth

**Fig. 4:**
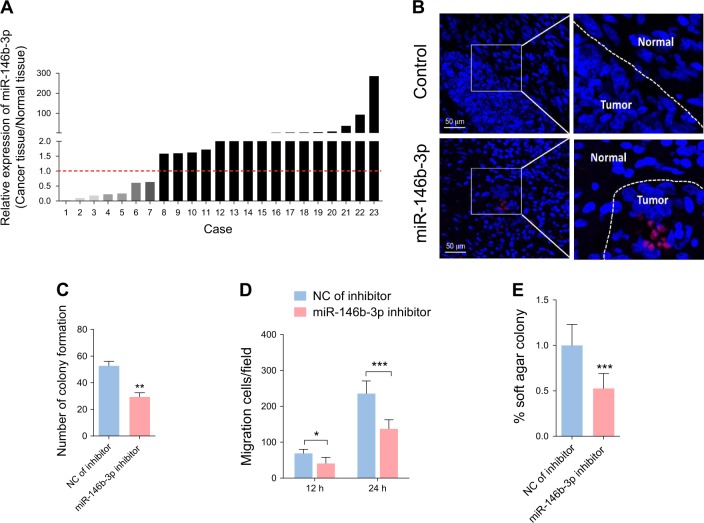
Inhibition of miR-146b-3p reduces cell proliferation, migration and anchorage-independent growth. (**a**) Expression of miR-146b-3p in 23 pairs of cervical cancer and adjacent non-cancerous tissues determined via qPCR. (**b**) Fluorescence in situ hybridization for miR-146–3p in cervical cancer and adjacent non-cancerous tissues. (**c**) Plate colony formation assay of SiHa cells transfected with a miR-146b-3p inhibitor for 48 h. (**d**) Transwell migration assay for cells treated as in (**c**). (**e**) Soft agar colony formation assay of cells treated as in (**c**)

miR-146b-3p expression was further examined in 23 paired clinical cervical cancer and normal tissues. miR-146b-3p levels were elevated in the majority of cervical cancer tissues (16/23) compared with paracancerous tissues (Fig. 4a). In situ hybridization confirmed the presence of higher amounts of miR-146b-3p in cervical cancer tissues (Fig. 4b). To further determine the potential contribution of miR-146b-3p to specific cell development processes, miR-146b-3p function was blocked with a specific inhibitor in SiHa cells and in cell colony formation, transwell migration and soft agar colony formation assays conducted. Our collective findings strongly suggest that miR-146b-3p inhibition leads to suppression of cell proliferation, migration and anchorage-independent growth (Fig. 4c–e).

### HPGD negatively regulates STAT3 and AKT signaling pathways

**Fig. 5:**
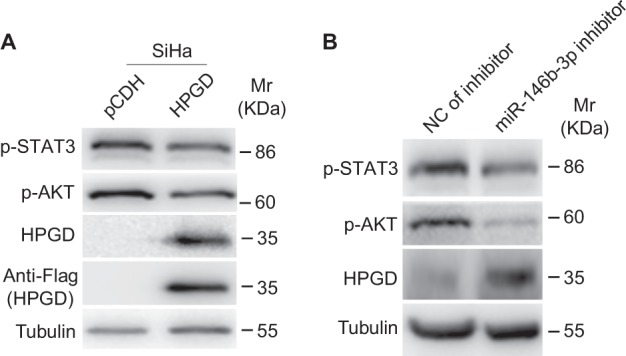
HPGD negatively regulates STAT3 and AKT activities (**a**) Western blot of SiHa cells transfected with HPGD with the indicated antibodies. (**b**) Western blot of SiHa cells transduced with a miR-146b-3p inhibitor with the indicated antibodies

**Fig. 6:**
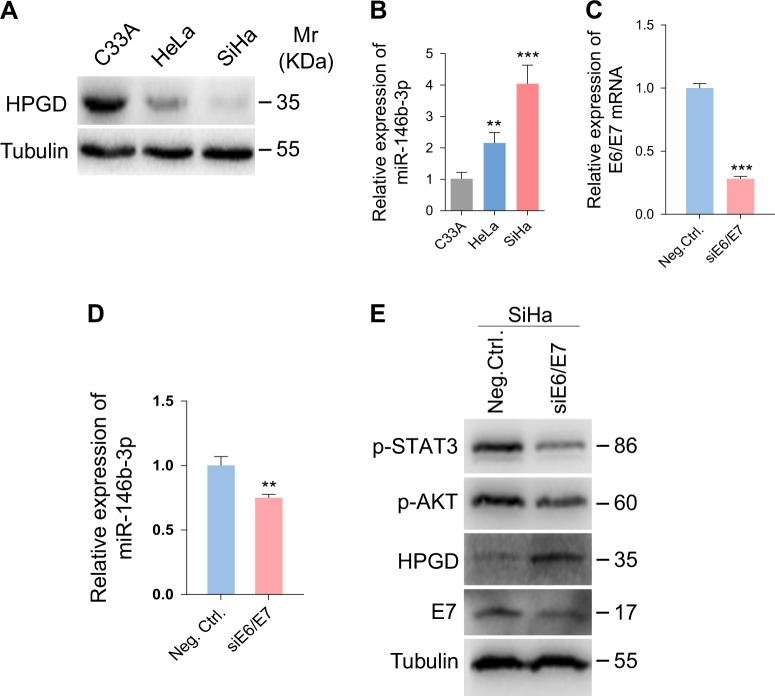
HPV E6/E7 promotes miR-146b-3p-mediated inhibition of HPGD (**a**) Western blot analysis of HPGD protein in C33A, HeLa and SiHa cells. (**b**) qPCR showing miR-146b-3p expression in C33A, HeLa and SiHa cells. (**c**) qPCR showing E6/E7 expression in SiHa cells with E6/E7 knockdown. (**d**) qPCR showing miR-146b-3p expression in SiHa cells with E6/E7 knockdown. (**e**) Western blot analysis of SiHa cells with E6/E7 knockdown with the indicated antibodies

Several studies have demonstrated that abnormal activation of STAT3 and AKT signaling pathways promotes cervical cancer cell proliferation, migration and cell transformation^35–37^. Accordingly, we focused on whether STAT3 and AKT pathways are involved in HPGD-mediated inhibition of cell proliferation, migration and anchorage-independent growth. Overexpression of HPGD induced a decrease in phosphorylated STAT3 and AKT levels (Fig. 5a and 6). Consistently, miR-146b-3p inhibition suppressed STAT3 and AKT activation (Fig. 5b), indicating that HPGD negatively regulates STAT3 and AKT activities.

### HPV E6/E7 promote miR-146b-3p-mediated inhibition of HPGD

To ascertain whether HPV infection plays a part in regulation of the miR-146b-3p/HPGD pathway, miR-146b-3p and HPGD expression patterns were examined in HPV-negative C33A, HPV18-positive HeLa and HPV16-positive SiHa cells. Our results showed a significant increase in miR-146b-3p and decrease in HPGD expression in HeLa and SiHa cells compared with C33A cells (Fig. 5a, b). The HPV oncoproteins E6 and E7 are the key factors that maintain the malignant phenotype of HPV-positive cancer cells^38^. To establish whether E6 and E7 exert regulatory effects on miR-146b-3p, these oncogenes were depleted with a mixture of siRNAs in SiHa cells (Fig. 5c), which induced a reduction in the miR-146b-3p level (Fig. 5d) and elevation of HPGD expression (Fig. 5e).

## Discussion

The HPGD gene located on chromosome 4 encodes a protein that is widely distributed in various mammalian tissues^39^. HPGD was initially characterized as a key enzyme responsible for biological inactivation of prostaglandins^25^ and subsequently shown to be involved in a number of human malignancies^22,40–42^. miR-620 is reported to up-regulate PGE2 expression by directly targeting HPGD, contributing to tumor radiation resistance^43^. An earlier study by He et al. demonstrated that miR-21 participates in tumorigenesis via targeting the HPGD/PGE2 signaling pathway^44^. Moreover, omega-3 polyunsaturated fatty acids (ω-3 PUFA) induce HPGD expression through inhibition of miR-26a and miR-26b, leading to suppression of human cholangiocarcinoma cell growth^45^. HPGD has been shown to regulate the 15-keto-PGE2/peroxisome proliferator-activated receptor-γ/p21 (WAF1/Cip1) signaling pathway to suppress hepatocellular carcinoma growth^46^. However, the role of HPGD in cancer biology has not been adequately addressed to date. Data from the current study disclosed that HPGD expression is remarkably down-regulated in cervical cancer tissues and its overexpression suppresses cell proliferation, migration and anchorage-independent growth. Our findings support the potential value of HPGD as a prognostic and therapeutic target for cervical cancer management.

miRNAs (18–25 nucleotides in length) act as negative regulators of gene expression by binding mRNA sequences to inhibit translation or destabilization of transcripts^47^. Accumulating evidence has shown that aberrant expression of specific miRNAs contributes to progression of multiple tumors, including cervical cancer^48^. For example, miR-494 is up-regulated in cervical cancer cells and promotes proliferation by directly targeting phosphatase and tensin homolog (PTEN)^49^. Conversely, miR-342–3p acts as a tumor suppressor and inhibits proliferation, migration and invasion in cervical cell lines through effects on FOXM1^50^. miR-491–5p is additionally down-regulated in cervical cancer tissues and suppresses cancer cell growth by targeting human telomerase reverse transcriptase (hTERT)^51^. On the other hand, miR-146b-3p is involved in progression and development of inflammation and arterial thrombosis^52^. Riesco-Eizaguirre et al. revealed abundant up-regulation of miR-146b-3p in papillary thyroid carcinoma, suggestive of its involvement in tumor progression^53^. In our investigation, frequent up-regulation of miR-146b-3p was evident in cervical cancer tissues and HPGD identified as its direct target. Moreover, suppression of miR-146b-3p inhibited cervical cancer cell proliferation, migration and anchorage-independent growth. The collective results indicate a critical role of miR-146b-3p in the pathogenesis of cervical cancer and support its application in tumor therapy.

Cervical cancer is responsible for 10–15% of cancer-related deaths in women worldwide, with HPV being the causative agent in over 99% cases. Although vaccines for HPVs and improvements in early screening have successfully reduced the mortality rate, cervical cancer is still considered a major public health problem in developing countries^1,54^. E6 and E7 encoded by high-risk HPVs are primary transforming viral proteins that regulate various cellular functions, such as proliferation, cell cycle, genomic instability and apoptosis^55^. Here, we further showed that knockdown of E6 and E7 trigger a decrease in miR-146b-3p and concomitant increase in HPGD. However, the precise underlying mechanisms remain to be established.

In conclusion, down-regulation of HPGD is associated with up-regulation of miR-146b-3p in cervical cancer. Moreover, overexpression of HPGD inhibits proliferation, migration and anchorage-independent growth in cervical cancer cells by inhibiting STAT3 and AKT activation. Our results clearly suggest that the miR-146b-3p/HPGD axis could serve as a promising prognostic and therapeutic target for cervical cancer management.

## Materials and Methods

### Cell culture transfection, reagents, and plasmids

HeLa, SiHa, C33A, and HEK293T cells were maintained in Dulbecco's modified Eagle's medium containing 10% fetal bovine serum at 37 °C with 5% CO_2_ in a humidified atmosphere. All cells were transfected using Lipofectamine 2000 Reagent (Invitrogen, Carlsbad, CA, USA) according to the manufacturer’s instructions. siRNAs, miRNA mimics and mutant miRNAs were synthesized by GenePharma (Suzhou, China) and miRNA inhibitors purchased from RiboBio (Guangzhou, China). The siRNA sequences are listed in Table 1. The respective HPGD 3’ UTR and HPGD CDS sequences were amplified via PCR and inserted into pGL3-Control plasmid (Promega, Madison, WI, USA). The 3 × Flag-HPGD construct was generated by inserting coding sequences into the lentiviral transferring plasmid pHAGE-CMV-MCSIzsGreen.

**Table 1:** Sequences of siRNAs

Gene	siRNA No.	Sequence of siRNA (5’ to 3’)
HPV16 E6/E7	si1	CCGGACAGAGCCCAUUACA
	si2	CACCUACAUUGCAUGAAUA
	si3	CAACUGAUCUCUACUGUUA

### Antibodies and western blotting

Anti-Flag, anti-phospho-AKT and anti-phospho-STAT3 antibodies were obtained from Cell Signaling Technologies (Beijing, China). Anti-GAPDH, anti-HPGD, anti-HPV16 E7 and anti-α-tubulin antibodies were acquired from Santa Cruz Biotechnology (Dallas, TX, USA). Western blot analysis was conducted as described previously^56,57^.

### Cell migration and plate colony formation assays

Cell migration and colony formation assays were conducted as described previously^58–60^.

### Soft agar assay

Cells (1 × 10^4^) suspended in 0.4% agarose (BD Biosciences) solution mixed with culture medium were seeded in six-well plates consisting of 0.8% agarose mixed with culture medium. Plates were incubated at 37 °C in the presence of 5% CO_2_ for 14 days. Five random fields were selected, photographed and cells were counted using NIH Image J software.

### Luciferase reporter assay

The luciferase reporter assay was conducted using the Promega dual-luciferase reporter assay system as described previously^61^.

### Reverse transcription and real-time quantitative PCR

RNA was extracted from cells using the RNA Isolator Total RNA Extraction Reagent (Vazyme Biotech Co., Ltd, Nanjing, China). Total RNA was reverse-transcribed with HiScript Q RT SuperMix (Vazyme Biotech Co., Ltd, Nanjing, China). Real-time quantitative PCR was performed using the AceQ qPCR SYBR Green Master Mix (Vazyme Biotech Co., Ltd, Nanjing, China). The primer sequences for PCR are presented in Table 2.

**Table 2:** Sequences of specific primers for qPCR (F, Forward; R, Reverse)

Target	Application	Primer
HPV16 E6/E7	RT-qPCR	F: 5′- CAATGTTTCAGGACCCACAGG -3′ R: 5′- CTCACGTCGCAGTAACTGTTG -3′
HPGD	RT-qPCR	F: 5′- CTCTGTTCATCCAGTGCGAT -3′ R: 5′- TCACTCCAGCATTATTGACCA -3′
ACLY	RT-qPCR	F: 5′- CTCCTCTGCTCGATTATGCACT -3′ R: 5′- CTCCCGAGTAAAGGACCCACA -3′
FAS	RT-qPCR	F: 5′- CGCCCACCTACGTACTGGCCTA -3′ R: 5′- GCTCCATGTCCGTGAACTGCT -3′
ELOVL6	RT-qPCR	F: 5′- TCTTCAGTATATTCGGTGCTC -3′ R: 5′- CTTAGCACAAATGCATAAGCC -3′
SCD1	RT-qPCR	F: 5′- CTACCTGCAAGTTCTACACC -3′ R: 5′- CAATGATCAGAAAGAGCCGTA -3′
β-actin	RT-qPCR	F: 5′- TTGCCGACAGGATGCAGAAGGA -3′ R: 5′- AGGTGGACAGCGAGGCCAGGAT -3′

### Immunohistochemistry (IHC)

Cervical cancer and normal tissue specimens provided by different hospitals were subjected to hematoxylin and eosin (H&E) and immunohistochemical staining. All human participants provided informed consent and all samples were anonymized. IHC was performed with specific antibodies as described previously^60,62^.

### Fluorescence in situ hybridization

Fluorescence in situ hybridization was performed as described in a previous report^63^. The sequences of the miR-146b-3p and control probes were 5′- ACCAGAACTGAGTCCACAGGGC-3′ and 5′- ACCACA ACTGAGTCCAGAGGGC -3′, respectively. The 5′ and 3′ ends of the probes were modified with digoxigenin.

### Statistical Analysis

All experiments were performed at least in triplicate, unless otherwise specified. Data are presented as means ± SD. Statistical analyses were conducted using the two-sided Student’s t-test. *P* values were calculated, and results considered statistically significant at *P* < 0.05.

## Electronic supplementary material

Supplementary Figure 1

Supplementary figure legends
