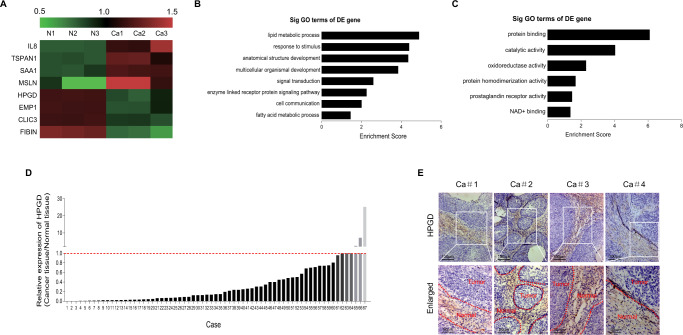# Correction: Down-regulation of HPGD by miR-146b-3p promotes cervical cancer cell proliferation, migration and anchorage-independent growth through activation of STAT3 and AKT pathways

**DOI:** 10.1038/s41419-023-05863-4

**Published:** 2023-06-27

**Authors:** Shuihong Yao, Jingyun Xu, Kaixuan Zhao, Pengxia Song, Qin Yan, Weifei Fan, Wan Li, Chun Lu

**Affiliations:** 1 State Key Laboratory of Reproductive Medicine, Nanjing Medical University, Nanjing, 211166 P. R. China; 2 Medical School, Quzhou College of Technology, Quzhou, 324000 P. R. China; 3 Key Laboratory of Pathogen Biology of Jiangsu Province, Nanjing Medical University, Nanjing, 211166 P. R. China; 4 Department of Microbiology, Nanjing Medical University, Nanjing, 211166 P. R. China; 5 Department of Hematology and Oncology, Department of Geriatric Lung Cancer Research Laboratory, Geriatric Hospital of Nanjing Medical University, Nanjing, 210024 P. R. China

Correction to: *Cell Death and Disease* 10.1038/s41419-018-1059-y, published online 17 October 2018

The original version of this article unfortunately contained an error in a figure 1. The corrected figure can be found below. The authors apologize for the error. The authors confirm that this correction does not affect either the results or the conclusions of the paper.