# Supplementary figures and images for: Down-regulation of HPGD by miR-146b-3p promotes cervical cancer cell proliferation, migration and anchorage-independent growth through activation of STAT3 and AKT pathways
Source: Cell Death Dis. 2018 Oct 17;9(11):1055. doi: 10.1038/s41419-018-1059-y (PMC6192999; doi:10.1038/s41419-018-1059-y)

**A**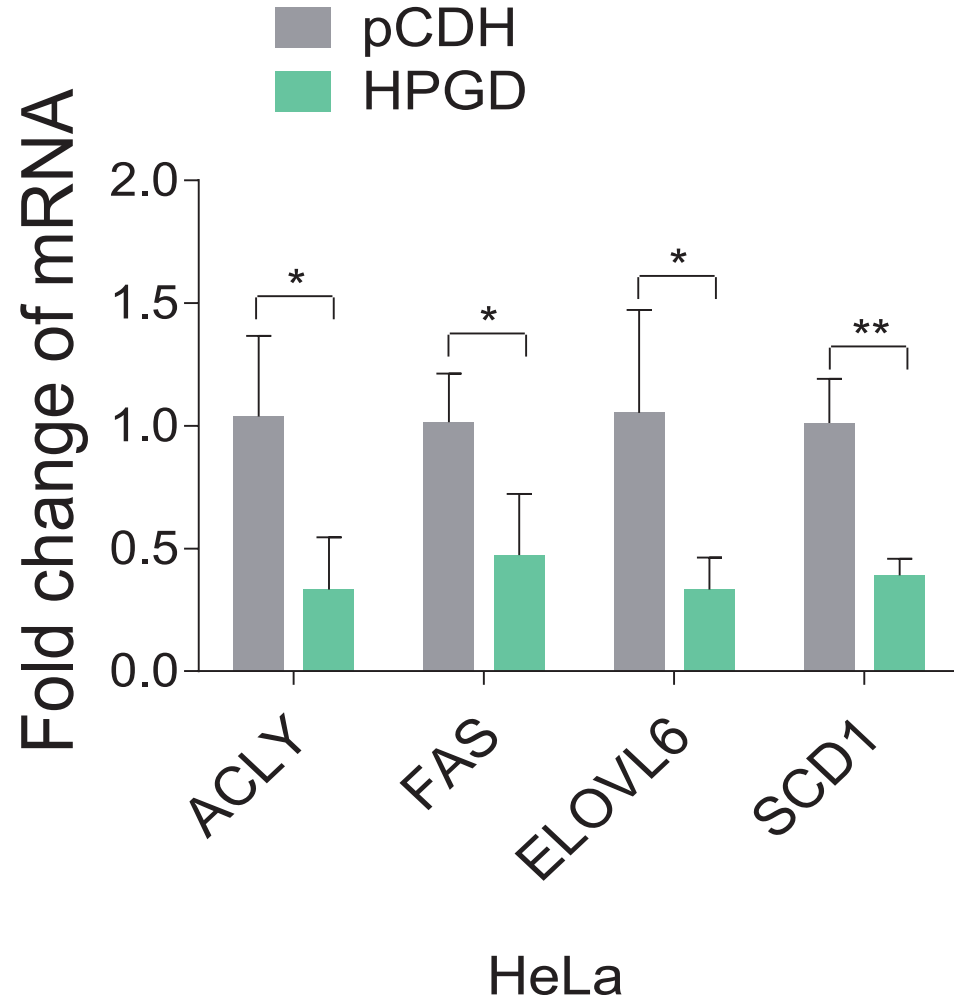**B**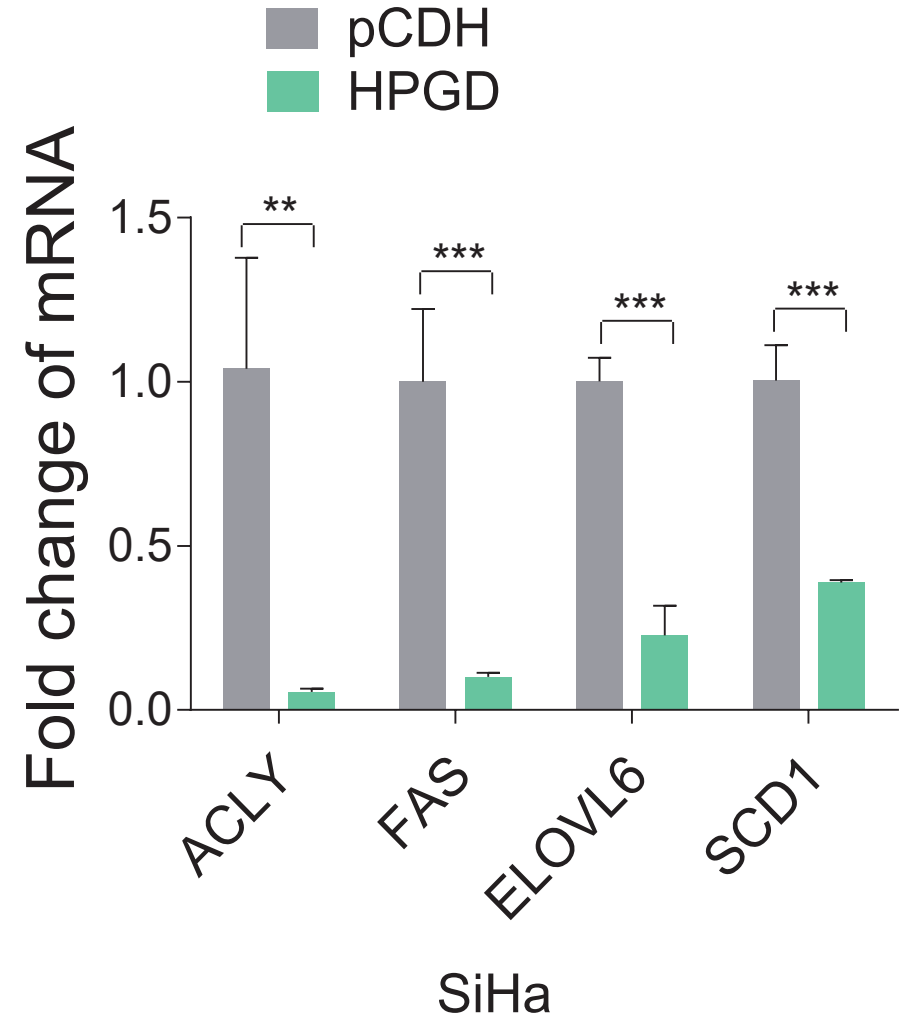

Supplementary Figure 1

Supplement: Supplementary file 1 — Supplementary Figure 1 [file 41419_2018_1059_MOESM1_ESM.pdf]
