# Supplementary material for: Down-regulation of HPGD by miR-146b-3p promotes cervical cancer cell proliferation, migration and anchorage-independent growth through activation of STAT3 and AKT pathways
Source: Cell Death Dis. 2018 Oct 17;9(11):1055. doi: 10.1038/s41419-018-1059-y (PMC6192999; doi:10.1038/s41419-018-1059-y)
Supplement: Supplementary file 2 — Supplementary figure legends [file 41419_2018_1059_MOESM2_ESM.docx]

**Supporting Information Legends**

**Supplementary Figure 1. HPGD inhibits *de novo* synthesis of fatty acids**

**(A)** qPCR results showing ACLY, FAS, ELOVL6 and SCD1 expression in HPGD-transduced HeLa cells.

**(B)** qPCR results showing ACLY, FAS, ELOVL6 and SCD1 expression in HPGD-transduced SiHa cells.

The quantified results are presented as means ± SD. Three independent experiments, each with four technical replicates, were performed. * *P* < 0.05,** *P* < 0.01, and *** *P* < 0.001 for Student's t-test.
